# A study to quantify surgical plume and survey the efficiency of different local exhaust ventilations

**DOI:** 10.1038/s41598-021-92859-9

**Published:** 2021-07-08

**Authors:** Ping-Chia Cheng, Ming-Hsun Wen, Wan-Lun Hsu, Po-Wen Cheng, Li-Jen Liao

**Affiliations:** 1 Department of Otolaryngology Head and Neck Surgery, Far Eastern Memorial Hospital, No. 21, Sec. 2, Nanya S. Rd., Banqiao Dist., New Taipei City, 220 Taiwan, ROC; 2 Genomics Research Center, Academia Sinica, Taipei, Taiwan, ROC; 3 Department of Electrical Engineering, Yuan Ze University, Taoyuan, Taiwan, ROC; 4 Medical Engineering Office, Far Eastern Memorial Hospital, New Taipei City, Taiwan, ROC

**Keywords:** Health care, Occupational health

## Abstract

This study aimed to compare the concentration of surgical smoke produced by different tissues and electric diathermy modes and to measure the effectiveness of various local exhaust ventilations. We compared the surgical plume concentration from different tissues and settings with a porcine tissue model. We also compared the efficiency of three local exhaust ventilations: (1) a desktop unit (Medtronic Rapid Vac), (2) a central evacuation system with ENT suction, and (3) a central evacuation system with a urethral catheter (PAHSCO Urethral Catheter). In the cutting setting, the skin tissue had a higher concentration of total suspended particulates (TPS), which were 1990 ± 2000 (mean ± SD, μg/m^3^), 6440 ± 3000 and 9800 ± 2300 at 15, 30 and 45 s, respectively (p < 0.05). In the coagulation setting, the adipose tissue had a higher concentration of TPS, which were 3330 ± 2600, 11,200 ± 5500 and 15,800 ± 7300, respectively (p < 0.05). We found that all three smoke extractors had more than 96% efficiency in clearing surgical smoke. With electric diathermy, skin tissue in the cutting model and adipose tissue in the coagulation mode will produce higher concentration of particles within surgical plumes. An electric surgical scalpel adapted with a urethral catheter is a simple and effective way to exhaust smoke in surgical operations.

## Introduction

Surgical smoke/plume is a term commonly associated with electrosurgery and cautery devices. Surgical staff may be exposed to surgical smoke, which may cause harm. Previous studies found that surgical smoke has a lifetime risk of carcinogenesis, and exposure to surgical smoke resulted in a cancer risk of approximately 117 × 10^–6^ for surgeons^1^. Surgical smoke can be divided into biological and nonbiological byproducts. Human papillomavirus (HPV) can be detected in surgical smoke generated by the electrosurgical unit and LASER during the treatment of laryngeal papilloma^2^ and genital infection^3^. Gloster et al. reported a higher prevalence of HPV localization in the nasopharynx of laser surgeons^4^. The Mayo Clinic found that otolaryngologists who performed procedures with CO_2_ LASER had an increased incidence of nasopharyngeal warts against age- and sex-matched controls, despite wearing surgical masks^5^. The latest reviews conclude that surgeons are at a risk for HPV exposure by inhalation of electrocautery and LASER-derived aerosols^6,7^. Nonbiological byproducts include polycyclic aromatic hydrocarbons (PAHs), cresols, phenol, benzene, toluene, xylene, aldehydes, hydrocyanic acid, carbon monoxide, and nitrile compounds^1^. These nonbiological byproducts are carcinogenic and harmful to the surgical team. Recent study showed that surgical smoke from human breast tissues is cytotoxic to human small airway epithelial cells in vitro^8^. The main carcinogens among surgical smoke are PAHs. In the meta-analyses by the International Agency for Research on Cancer (IARC), lung cancer was the most common PAH-related cancer^9^. The United States Environmental Protection Agency (EPA) listed 16 PAHs as priority pollutants^10^.

Surgical energy devices produce a spectrum of aerosolized particles. The common aerodynamic equivalent diameter (AED) of particles produced during surgery is between 0.05 and 25 μm, and the AED of particles produced by electrocautery is between 0.3 and 0.5 μm^1^, with the possibility of transmission through the surgical mask^11^. Therefore, high-efficiency particulate air respirator masks are better for the intraoperative use of surgical energy devices in the COVID-19 pandemic^5^.

Besides the surgical mask, the surgical smoke capture systems, including local exhaust ventilators (LEVs) and central evacuation systems, are also important. The central evacuation systems remove smoke through a filter and return ambient air to the operating room^12^. The LEV uses a desktop suction pump connected with an electrosurgical unit pencil. Severe acute respiratory syndrome coronavirus 2 (SARS-CoV-2) is the pathogen of COVID-19. The size and deposition of SARS-CoV-2 could be provided for reference^13^. SARS-CoV-2 is approximately 50 to 200 nm in diameter and has been shown to remain viable in aerosols^14^. Several LEVs were developed for open surgery during the COVID-19 pandemic^15^. A previous study evaluated the effect of LEV with a significant reduction in surgical smoke^16^. However, no study has compared the surgical smoke produced by different electric diathermy mode, and the effectiveness of various smoke evacuation systems requires a robust assessment^12^. Hill et al. used porcine tissue model for evaluating the tissue destroyed during electric diathermy, they found that there’s no difference between porcine tissue model and human tissue sample^17^. Considering their finding, the accessibility of porcine tissue and ethical issues with human tissue, we chose the porcine tissue for this study. We used the electric diathermy with cutting or coagulation mode over skin, subcutaneous fat, and muscular tissue in order to simulate most surgery. We chose the electric diathermy with coagulation mode over muscular tissue, which was the most commonly encountered during head and neck surgery, to evaluate the efficiency of LEV.

The purpose of this study was to measure the surgical smoke produced by different tissues and to compare the effectiveness of different local exhaust ventilators.

## Methods

**Figure 1:**
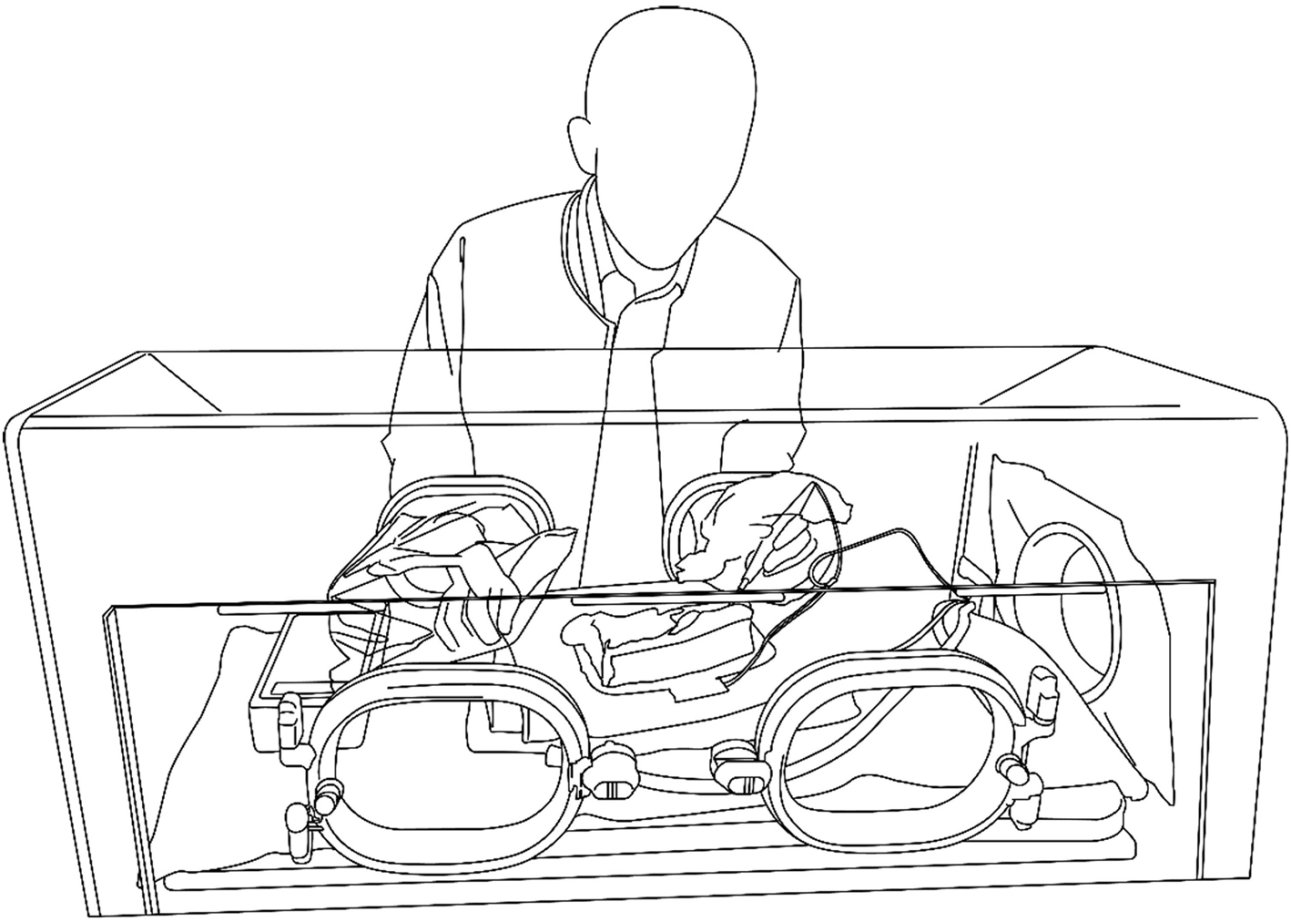
The setting of experiment. Porcine tissue was placed at the center of a closed room (Infant Incubator ATOM V-2100G). We used MET ONE AEROCET 831 (placed on the right side) to measure surgical smoke with different aerodynamic equivalent diameters (AEDs) of particle matter (less than 1 μm, 1–2.5 μm, 2.5–4 μm, 4–10 μm, larger than 10 μm and TSP). The authors thank Cheng-Han, Li for this drawing.

The study was performed during 2020. We used porcine tissue to simulate the human body, with electric diathermy as the surgical equipment. The size of the porcine tissue was approximately 20 × 10 × 2 cm and is composed of skin, subcutaneous fat, and muscular tissue. All experiments were performed in a closed room (Infant Incubator ATOM V-2100G, with size 100 × 58.5 × 134.5 cm) (Fig. 1). The porcine tissue was placed on the central. A handheld particle counter (Met One Aerocet 831) was used to measure surgical smoke with different AEDs of particulate matter (PM), including less than 1 μm, 1–2.5 μm, 2.5–4 μm, 4–10 μm, larger than 10 μm and total suspended particulates (TSP). The particle counter was placed on the right side of porcine tissue, with a distance of approximately 15 cm. The experiment was composed of 2 sections. First, we used cutting or coagulation diathermy over different tissues of a porcine model and recorded the concentration (μg/m^3^) of different PM. Second, we used coagulation diathermy over muscular tissue of a porcine model with different LEVs to measure the efficiency.

### Comparisons of surgical smoke concentrations from different tissues

We used electric diathermy (coagulation or cutting mode, 30 watts, Valleylab Force 2) with an electric scalpel (Valleylab Rocker Switch Pencils) over the skin, subcutaneous fat, and muscular tissue for 15, 30 and 45 s, each five times. We recorded the average concentration of different PM.

### Comparisons of the efficiency of different local exhaust ventilations (LEVs)

**Figure 2:**
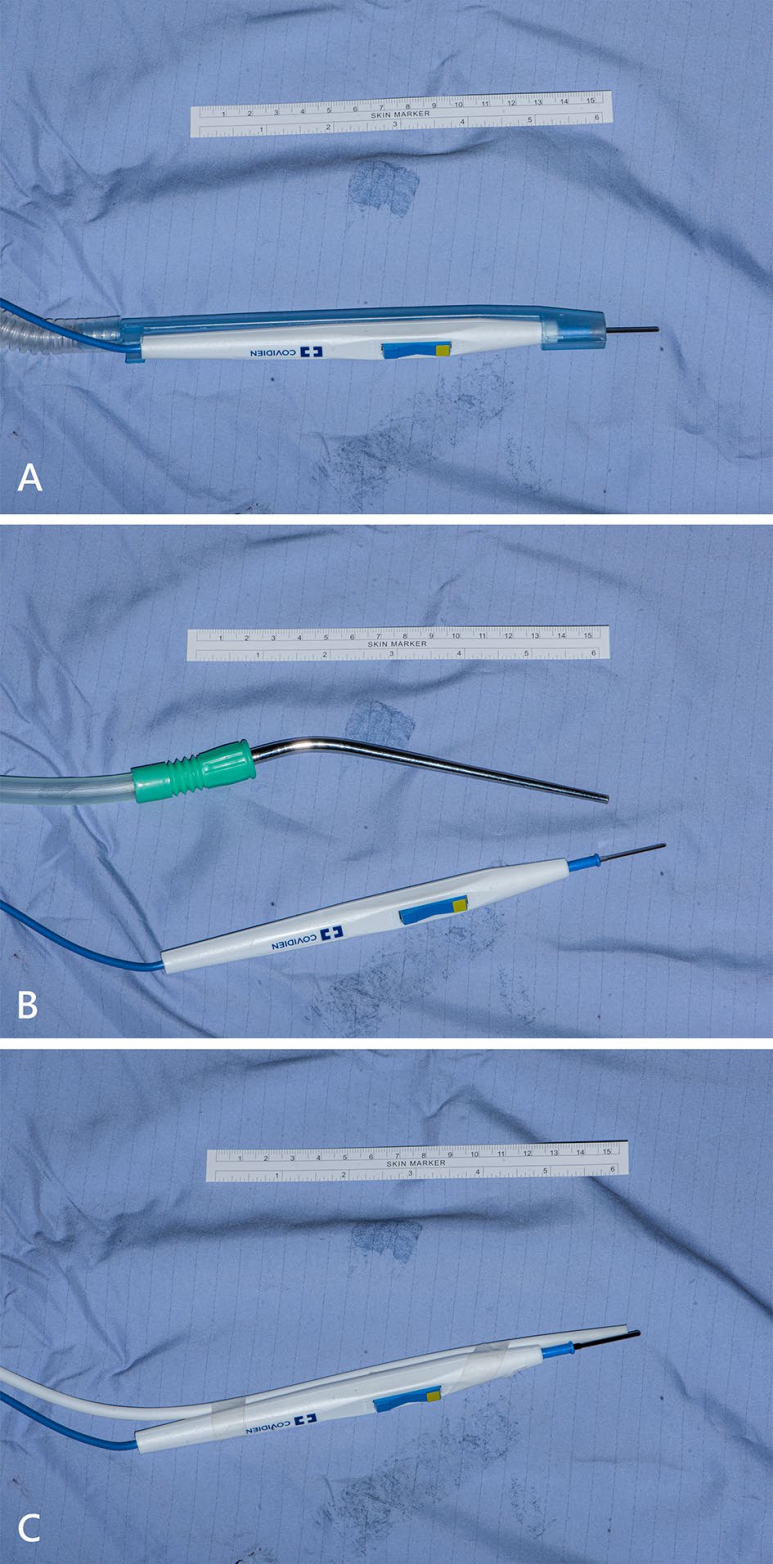
Preparation of various local exhaust ventilation. (**A**) Desktop unit: Medtronic Rapid Vac with the electric scalpel suction adapter (inner diameter 10.0 mm with tip flow 1250 L/min). (**B**) Surgical suction (ENT suction, inner diameter 1.5 mm with tip flow 40.6 L/min). (**C**) PAHSCO urethral catheter (inner diameter 2.5 mm with tip flow 29 L/min).

**Figure 3:**
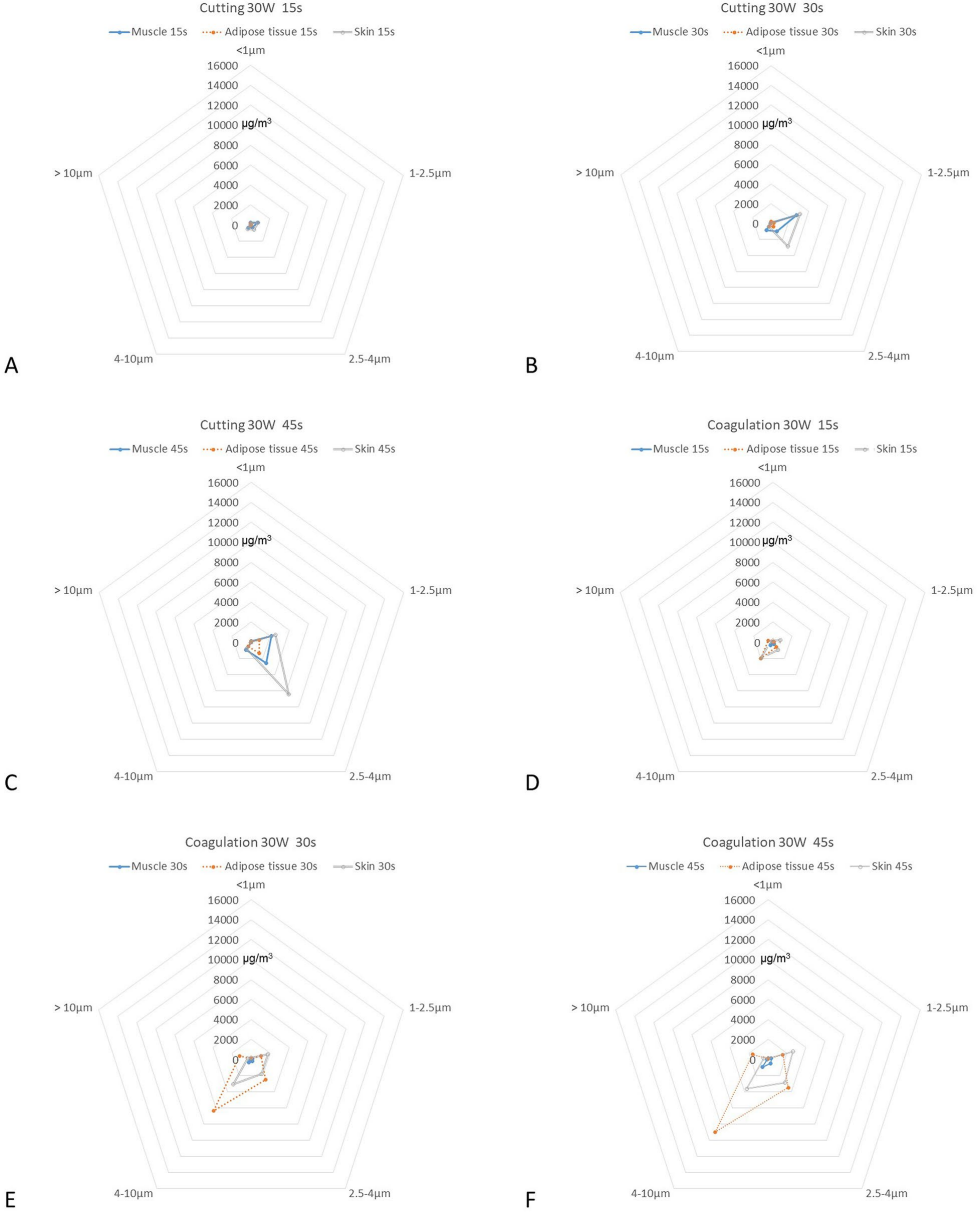
The concentration of different types of particulate matter among different tissues in porcine model with cutting (**A**–**C**) and coagulation (**D**–**F**) modes. The measured average smoke concentration increased gradually with time. Skin tissue in cutting mode and adipose tissue in coagulation mode would produce higher smoke concentrations.

We compared the efficiency of the following instruments: (1) desktop unit (Medtronic Rapid Vac with inner diameter 10 mm and tip flow 1250 L/min, Fig. 2A), (2) central unit with surgical suction (ENT suction, inner diameter 1.5 mm with tip flow 40.6 L/min, Fig. 2B), and (3) central unit with urethral catheter (PAHSCO urethral catheter with inner diameter 2.5 mm and the tip flow is 29 L/min, Fig. 2C). First, we measured the background particle concentration. Then, we used electric diathermy without and with LEV to evaluate the efficiency of LEV. We used coagulation diathermy (coagulation mode, 30 watts) over muscular tissue for 15, 30 and 45 s, each five times, and recorded the average concentration of different PM. We defined the efficiency of LEV as [(concentration without LEV-background concentration) − (concentration with LEV-background concentration)]/(concentration without LEV-background concentration). The results are shown as the average five times for different PMs. We also measured the air flow at the tip of each LEV.

### Statistical analysis

We used STATA software, version 12.0 (Stata Corporation, College Station, TX), for the data analysis. Continuous variables are expressed as the mean ± SD (standard deviation). We used linear regression analysis to test different tissues and time durations for the production of TSP. Differences among ß-regression coefficients were considered significant when p < 0.05.

### Ethical approval and consent

Ethical approval was not required for this study because no humans or live animals were involved in the study process.

## Results

With our porcine tissue model, we found that the concentrations of each particle increased gradually with prolonged diathermy duration (Table 1 and Fig. 3A–F). We found that the TSP had a positive correlation with the duration of diathermy in both cutting and coagulation modes (p < 0.01, Table 2).

**Table 1:** The concentration [mean (SD), μg/m^3^] of various aerodynamic equivalent diameters (AEDs) of particle in different tissues of the porcine model using coagulation and cutting modes under 30 W power.

AEDs of particle	< 1 μm	1–2.5 μm	2.5–4 μm	4–10 μm	> 10 μm	TSP
**Cutting mode**						
Muscle						
15 s	227 (40)	709 (390)	300 (230)	380 (420)	46 (41)	1660 (400)
30 s	99 (55)	2680 (1000)	936 (580)	816 (750)	76 (75)	4610 (970)
45 s	124 (87)	2140 (1200)	2570 (2800)	924 (640)	37 (25)	5790 (3700)
Adipose						
15 s	97 (40)	67 (44)	23 (12)	46 (43)	8 (17)	240 (81)
30 s	181 (51)	328 (430)	364 (660)	342 (420)	28 (15)	1240 (1500)
45 s	136 (44)	841 (1100)	1340 (2500)	500 (400)	39 (44)	2850 (3700)
Skin						
15 s	153 (84)	754 (1200)	574 (890)	482 (600)	24 (29)	1990 (2000)
30 s	56 (18)	3090 (340)	2840 (2900)	422 (380)	29 (26)	6440 (3000)
45 s	44 (18)	2520 (420)	6410 (2100)	805 (440)	25 (11)	9800 (2300)
**Coagulation mode**						
Muscle						
15 s	42 (42)	67 (130)	178 (210)	331 (390)	31 (37)	650 (780)
30 s	77 (44)	130 (100)	188 (110)	376 (240)	35 (29)	807 (400)
45 s	99 (31)	363 (220)	479 (370)	954 (790)	84 (66)	1980 (1500)
Adipose						
15 s	76 (51)	163 (160)	582 (670)	2030 (1600)	483 (430)	3330 (2600)
30 s	100 (41)	1060 (770)	2470 (1400)	6330 (3000)	1200 (640)	11,200 (5500)
45 s	109 (30)	1550 (450)	3470 (1700)	8990 (4600)	1630 (920)	15,800 (7300)
Skin						
15 s	151 (55)	808 (730)	952 (640)	1920 (1600)	261 (370)	4090 (2700)
30 s	145 (74)	1760 (990)	1760 (1500)	3040 (3900)	331 (690)	7040 (6300)
45 s	77 (31)	2620 (470)	2870 (1600)	3630 (5600)	428 (932)	9630 (7400)

*TSP* total suspended particulates.

**Table 2:** Multiple linear regression analysis of the relation between tissue and duration of electrical diathermy with TSP.

TSP	ß-Coef	Std	p-value	[95% Conf. Interval]	
**Cutting mode**					
Tissue					
Muscle	Reference				
Skin	2056.44	876.26	0.024	286.80	3826.08
Adipose	− 2577.12	876.26	0.005	− 4346.76	− 807.48
Time (s)	161.81	29.21	0.000	102.82	220.80
**Coagulation mode**					
Tissue					
Muscle	Reference				
Skin	5775.76	1753.46	0.002	2234.58	9316.94
Adipose	8940.21	1753.46	0.000	5399.03	12,481.39
Time (s)	214.19	58.45	0.001	96.15	332.23

*TSP* total suspended particulates.

### Cutting setting

In cutting mode, we found that the skin tissue had a higher concentration of TSP. The concentrations for TSP were 1990 ± 2000 (mean ± SD, μg/m^3^), 6440 ± 3000 and 9800 ± 2300 at 15, 30 and 45 s, respectively, for skin tissue. The concentrations were 240 ± 81, 1240 ± 1500 and 2850 ± 3700 for adipose tissue and 1660 ± 400, 4610 ± 970 and 5790 ± 3700, respectively, for muscular tissue (Table 1). After adjusting for duration, we found that skin (ß-Coef. = 2056.44, p-value = 0.024) had a higher TSP concentration than muscle and adipose tissue (Table 2). The major AED of particle was within 2.5–4 μm (Fig. 3C).

### Coagulation setting

In coagulation mode, the concentrations for TSP were 4090 ± 2700 (mean ± SD, μg/m^3^), 7040 ± 6300 and 9630 ± 7400at 15, 30 and 45 s, respectively, for skin tissue. The concentrations were 3330 ± 2600, 11,200 ± 5500 and 15,800 ± 7300 for adipose tissue and 650 ± 780, 807 ± 400 and 1980 ± 1500 for muscular tissue, respectively (Table 1). After adjusting for duration, we found that adipose tissue (ß-Coef. = 8940.21, p-value < 0.01) had a higher TSP concentration than muscle and skin tissue (Table 2). The major AED of particle was within 4–10 μm (Fig. 3F). We also found that the skin tissue had a high concentration at 15 s, but adipose tissue had a higher concentration at 30 and 45 s (Table 1).

### Efficiency of different local exhaust ventilations

The efficiency results of different LEVs are shown in Table 3. The measured air flow at the tip of each LEV was different from the document. The measured flow of the Medtronic Rapid Vac, surgical suction, and urethral catheter was 50.6, 40.6, and 36.9 L/min, respectively. We found that the efficiency of three different LEVs was near 100%. The lowest efficiency (96%) was noted after coagulation for 45 s with Medtronic Rapid Vac. The efficiency of LEV was 96–99% for < 1 μm and 99–100% for > 2.5 μm.

**Table. 3:** Comparison of smoke evacuation efficiency of different local exhaust ventilations under coagulation (power 30 W) with muscular tissue.

Local exhaust ventilation	Medtronic rapid vac (Fig. 2A)	Surgical suction (Fig. 2B)	Urethral catheter (Fig. 2C)
Measured flow at tip (L/min)	50.6	40.6	36.9
**Efficiency (%), mean (SD)**			
< 1 μm			
15 s	100% (0.8%)	100% (0.8%)	100% (0.2%)
30 s	100% (0.3%)	100% (0.3%)	100% (0.2%)
45 s	96% (6.2%)	98% (4.2%)	99% (0.8%)
1–2.5 μm			
15 s	99% (1.8%)	100% (0.5%)	100% (0.3%)
30 s	100% (0.7%)	99% (1.1%)	99% (0.5%)
45 s	98% (2.3%)	99% (2.6%)	99% (0.7%)
2.5–4 μm			
15 s	100% (0.9%)	98% (4.3%)	100% (0.3%)
30 s	100% (0.4%)	100% (1.1%)	100% (0.2%)
45 s	99% (1.3%)	99% (1.1%)	100% (0.3%)
4–10 μm			
15 s	100% (0.6%)	98% (4.3%)	100% (0.3%)
30 s	100% (0.2%)	99% (2.7%)	100% (0.5%)
45 s	99% (0.8%)	99% (1.1%)	100% (0.1%)
> 10 μm			
15 s	100% (0.9%)	100% (0.9%)	100% (0.0%)
30 s	100% (0.8%)	97% (4.7%)	100% (0.0%)
45 s	99% (1.7%)	100% (0.1%)	100% (0.1%)
TSP			
15 s	100% (0.8%)	99% (3.7%)	100% (0.3%)
30 s	100% (0.2%)	99% (2.0%)	100% (0.3%)
45 s	99% (1.5%)	99% (1.5%)	100% (0.3%)

*TSP* total suspended particulates.

## Discussion

During operations with energy devices, surgical staff may be exposed to surgical smoke that causes health concerns. How to easily detect surgical smoke and prevent inhalation of aerosolized particles is our main target. A porcine tissue model has been used to assess surgical smoke in plastic surgery theatres. On average, the daily smoke production was equivalent to 27–30 cigarettes^17^. With the assistance of a handheld particle counter, we can easily measure the different PM of surgical smoke. Our report is the first study to quantify the concentration of surgical smoke in different tissues and diathermy setting with a porcine model.

Our study reveals that the concentration varies in different tissues and diathermy settings. Skin incision is usually the first step in open surgery. According to our study, under the cutting mode, skin tissue will produce higher concentration of particles within surgical plumes than adipose and muscular tissue (Tables 1, 2). Therefore, we suggest that surgeons use cold knives for skin incisions with selective use of coagulation over the bleeding point. We also found that adipose tissue had higher surgical smoke with coagulation mode (Tables 1, 2). Thus, during the operation of lipoma or lymphoareolar tissue, we should be more careful with the use of the coagulation mode. However, after skin incision in open surgery, we usually encounter lymphoareolar tissue with capillaries or small vessels, and diathermy with coagulation mode is inevitable. In such situations, local exhaust ventilation is more helpful.

We also developed an easy-setting electrocautery smoke evacuation device with a urethral catheter beside the surgical pencil, which is convenient and disposable (Fig. 2C). Based on our study, we noted that three different LEVs were all effective for the removal of surgical smoke. The efficacies of the three different LEVs were all near 100%. Even with the assistance of simple ENT suction, we can easily exhaust surgical smoke and achieve an environment with a low concentration of PM. However, if we used ENT suction for the removal of surgical smoke, another assistant was needed to hold the suction, and additional suction may be necessary to clean blood in the surgical field. Both the Medtronic Rapid Vac system and urethral catheter adapted with an electrosurgical pencil can reduce surgical smoke without assistance. The Medtronic Rapid Vac system has its own desktop filter, but the adapted urethral catheter needs a central high-efficiency particulate air filter (HEPA) system.

COVID-19 is a pandemic in 2020, and it causes serious morbidity and mortality worldwide. Protection from electrosurgery-related smoke is recommended during the pandemic^15^. Therefore, this topic is more important. Several smoke evacuation devices have been reported^15,18^. A previous study evaluated the effect of LEV with a significant reduction in surgical smoke^16^. The average mass concentration of surgical smoke with PlumeSafe®Turbo was less than that in background, which achieved efficiency with 100%. In our study, we developed another simple and effective method with a disposable urethral catheter to exhaust smoke in surgical operation by using this synchronous simple smoke evacuator in addition to the electric scalpel. The efficiency of the adapted urethral catheter was also around 99–100%. Although our study results were not strong enough to make solid conclusions or recommendations about how we should practice surgery, it still formed the foundation for further work.

## Limitation

There are some limitations in this study. First, we used the porcine model for this experiment instead of human tissue. Dead animal tissue is not represented as a component of live human tissue. Second, the efficiency of LEV was only tested in muscular tissue under coagulation mode. Further study can be evaluated with cutting mode over different tissue. Please forgive us that we can’t do further tests due to the serous outbreak of COVID-19 on Taiwan now. Third, surgical smoke is associated with not only electric scalpels but also other electrical devices. LASER and other power instruments were not included in our study. Airborne byproducts can also be generated by ultrasonic aspirators/scalpels, high-speed drills, burrs and saws. Further study is suggested to focus on the surgical smoke produced by these instruments.

## Conclusion

With electric diathermy, skin tissue in cutting mode and adipose tissue in coagulation mode will produce higher concentration of particles within surgical plumes. We developed a simple and effective method with a disposable catheter to exhaust smoke during surgical operations by using a synchronous simple smoke evacuator near the electric scalpel. Although our study results were not strong enough to make solid conclusions or recommendations about how we should practice surgery, it still formed the foundation for further work.

## Data Availability

Datasets from this study are publicly available.
